# Hormonal and Inflammatory Responses to Hypertrophy-Oriented Resistance Training at Acute Moderate Altitude

**DOI:** 10.3390/ijerph18084233

**Published:** 2021-04-16

**Authors:** Cristina Benavente, Josefa León, Belén Feriche, Brad J. Schoenfeld, Juan Bonitch-Góngora, Filipa Almeida, Sergio Pérez-Regalado, Paulino Padial

**Affiliations:** 1 Department of Physical Education and Sport, Faculty of Sport Sciences, University of Granada, 18011 Granada, Spain; cbenavente@ugr.es (C.B.); mbelen@ugr.es (B.F.); juanbonitch@ugr.es (J.B.-G.); filipaalmeida@ugr.es (F.A.); serperel284@gmail.com (S.P.-R.); ppadial@ugr.es (P.P.); 2 Clinical Management Unit of Digestive System, San Cecilio Hospital, ibs.GRANADA, 18016 Granada, Spain; 3 Department of Health Sciences, CUNY Lehman College, Bronx, NY 10468, USA; bradschoenfeldphd@gmail.com

**Keywords:** strength, cytokines, miRNA, terrestrial altitude, hypoxia

## Abstract

This study investigated the effect of a traditional hypertrophy-oriented resistance training (R_T_) session at acute terrestrial hypoxia on inflammatory, hormonal, and the expression of miR-378 responses associated with muscular gains. In a counterbalanced fashion, 13 resistance trained males completed a hypertrophic R_T_ session at both moderate-altitude (H; 2320 m asl) and under normoxic conditions (N; <700 m asl). Venous blood samples were taken before and throughout the 30 min post-exercise period for determination of cytokines (IL6, IL10, TNFα), hormones (growth hormone [GH], cortisol [C], testosterone), and miR-378. Both exercise conditions stimulated GH and C release, while miR-378, testosterone, and inflammatory responses remained near basal conditions. At H, the R_T_ session produced a moderate to large but nonsignificant increase in the absolute peak values of the studied cytokines. miR-378 revealed a moderate association with GH (r = 0.65; *p* = 0.026 and r = −0.59; *p* = 0.051 in N and H, respectively) and C (r = 0.61; *p* = 0.035 and r = 0.75; *p* = 0.005 in N and H, respectively). The results suggest that a R_T_ session at H does not differentially affect the hormonal, inflammatory, and miR-378 responses compared to N. However, the standardized mean difference detected values in the cytokines suggest an intensification of the inflammatory response in H that should be further investigated.

## 1. Introduction

Over the past decade, resistance training (R_T_) under hypoxic conditions has become a topic of great interest due to its potential beneficial effect on mechanisms related to strength and muscle mass development [1,2,3]. An elevation in systemic hormonal production, cell swelling, and alterations in local myokines, among others, are considered anabolic factors from the associated exercise-induced metabolic stress [4]. The combination of strength exercise and hypoxic conditions results in a greater desaturation of muscular oxygen [1], which in turn is linked to an increase in anaerobic metabolism and therefore to an increase in the production of metabolites [2,5], enhancing the hypertrophic response [4]. In particular, increases in circulating ions (Na^+^, Cl^−^, Ca^2+^, and H^+^) [6] and blood lactate [1,6,7,8] have been reported after acute R_T_ exercise at moderate and severe hypoxia.

**Figure 1:**
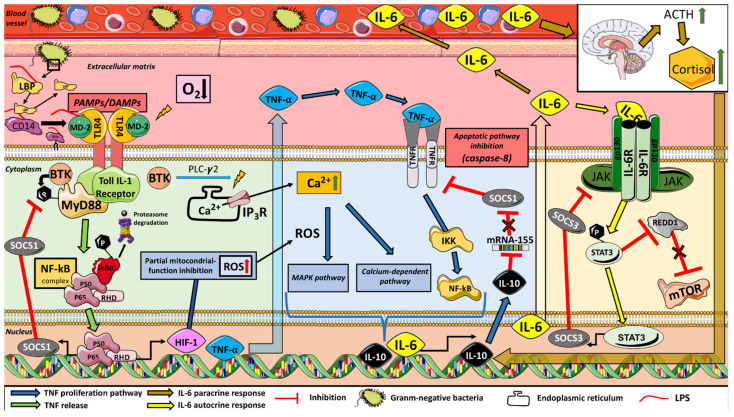
The acute inflammatory response to exercise in a hypoxic environment upregulates several signaling pathways that modulate cytokine release in an autocrine and paracrine activity of IL-6. LBP: Lipopolysaccharide binding protein; CD14: Cluster of differentiation 14; MD-2: Lymphocyte antigen 96; TLR4: Toll-like receptor 4; PAMPs: Pathogen-associated molecular patterns; DAMPs: Damage-associated molecular patterns; BTK: Bruton’s tyrosine kinase; MyD88: Myeloid differentiation primary response 88; PLC-γ2: Phospholipase C gamma2; NF-κB: Nuclear factor kappa B; SOCS: Suppressors of cytokine signaling; TNF-α: Tumor necrosis factor; TNFR: Tumor necrosis factor receptor; HIF-1: Hypoxia-inducible factor-1; ATP: Adenosine triphosphate; MAPK: Mitogen activated protein kinases; ROS: Reactive oxygen species; IKK: I kappa B kinase; AP-1: Activator protein 1; IL-10: Interleukin-10; IL-6: Interleukin-6; IL-6R: Interleukin-6 receptor; gp130: Glycoprotein 130; JAKs: Janus kinases; STATs: Signal transducer and activator of transcription proteins; REDD1: Regulated in development and DNA damage response 1; mTOR: Mammalian target of rapamycin; P: Phosphate; ACTH: Adrenocorticotropic hormone.

The interaction between metabolic stress and ion flow during exercise under hypoxic conditions also involves inflammatory and immune responses (Figure 1). In this context, circulating pro- and anti-inflammatory cytokines (such as IL-6, IL10 or TNFα) can provide useful insights due to their putative regulation of T-cell differentiation, both controllers of muscle regeneration [9]. Indeed, IL-6 is a signaling agent that activates the mTOR pathway, that regulates mRNA translation [10] and myogenesis [11]. Furthermore, the IL-6 response can be enhanced by the progressive inhibition of IL-10 on the apoptotic pathway of TNFα [12], favoring the activity of IL-6. Hypoxia exposure itself has been shown to increase circulation of pro-inflammatory cytokines at rest and during high intensity metabolic [13] and resistance exercise [14] compared to that at sea level. Previous studies suggest that the positive acute simulated hypoxia-induced hypertrophy response after a R_T_ session seems to activate satellite cell proliferation through the increase of IL-6/activating signal transducer and activator of transcription 3 (STAT3) [14]. This inflammatory response plays an essential role in skeletal muscle repair and remodeling after exercise-induced muscle damage [15]. However, the effect of acute or chronic terrestrial hypoxia or functional hypertrophy training session on the inflammatory response related to the activation of the myogenesis pathway has not been investigated.

Genetic predisposition is one of the most influential factors in the individual variability of the metabolic and inflammatory responses to resistance exercise [16]. In particular, recent research has focused on investigating post-transcriptional regulators via the role of microRNAs (miRNAs) in regard to their effect on athletes’ muscle response after R_T_. Important biological processes in muscle, such as growth, development, metabolic adaptation, and repair are regulated by a variety of miRNAs (e.g., miR-378, miR-1, miR-133a, miR-206, miR-208a, miR-208b, and miR-499). In addition, muscle mass gain after R_T_ in healthy individuals has been positively related to certain miRNAs such as miR-378 [16]. The overexpression of miR-378 increases the transcriptional activity of the myogenic differentiation factor (MyoD), purportedly by repressing its antagonist (MyoR) [17], which in turn may enhance processes related to muscle hypertrophy [14]. Therefore, local muscle protein expression and synthesis are apparently regulated by miRNAs [18]. The muscle protein regulatory capacity of miRNAs together with the potential effect of the R_T_ stress on muscle growth mechanisms may share a symbiotic relationship, which could be altered under hypoxic conditions.

The aims of this study were twofold: (1) To compare the acute effects of a hypertrophy-oriented R_T_ session under normoxia and terrestrial moderate hypoxia on exercise-induced inflammatory cytokines and anabolic hormones associated with muscular adaptations; and (2) to establish the influence of acute altitude on the potential relationship between miR-378 and these related variables. We hypothesized that the acute hypertrophy-oriented R_T_ session would increase the inflammatory, hormonal, and miR-378 responses, and that hypoxia would amplify these results.

## 2. Materials and Methods

### 2.1. Experimental Approach to the Problem

The experimental procedure was detailed in a previous study [19]. Briefly, a repeated measures design was used to compare the inflammatory, hormonal, and miR-378 responses throughout the initial 30 min post-R_T_ period at acute moderate-altitude (H; 2320 m asl) and under normoxic conditions (N; <700 m asl). In a counter-balanced order, participants performed two traditional hypertrophic-oriented R_T_ sessions under both environmental conditions. One week before the first R_T_ session, participants were required to complete a preliminary session to determine their 10-repetition maximum (RM) load in each exercise at a normoxic environment. Then, 72 h before the start of the study and after 48 h of rest at N, participants attended the laboratory for anthropometric (height (Seca 202, Seca Ltd., Hamburg, Germany), body mass (Tanita BC 418 segmental, Tokyo, Japan)) and resting blood sample testing.

During the experimental period, participants fasted after midnight of the day before each training session. They were provided with a meal replacement supplement (610 calories; 31% protein, 41% carbohydrate, and 12% fat) 1.5 h prior to the start of the corresponding warm-up. Testing sessions were conducted at the same time of day, at a temperature of ~22 °C and ~60% humidity, or ~22 °C and ~28% humidity for N and H conditions, respectively. Participants travelled by car to the altitude training center (32 km) to perform each training session. Arrivals to altitude occurred ~30 min before the training session and participants returned to normoxia after completing the session. Arterial oxygen saturation (SaO_2_; Wristox 3100; Nonin, Plymouth, MN, USA) was evaluated before the start of the warm-up of each training session to test the H condition. Participants displayed a mean SaO_2_ value of 94.2 ± 1.3 and 98.0 ± 1.5% in H and N, respectively (*p* < 0.001).

### 2.2. Subjects

Thirteen male volunteers (age: 22.31 ± 2.59 years; height: 178.31 ± 4.96 cm; body mass: 76.92 ± 9.17 kg) participated in the study. All participants had participated in a resistance training regimen for a minimum of 3 times per week for at least 12 months. Subjects had no health or muscular disorders, reported to be free from consumption of any agents associated with increased muscle size during the previous month, and had not been exposed to altitudes above 1500 m asl for more than 3–4 consecutive days for at least two months before the study onset. Participants lived at a low altitude to ensure that responses were specific to acute hypoxia exposure. This study was approved by the Local University Research Ethics Committee and conducted in accordance with the Helsinki Declaration. Informed written consent was obtained from all participants prior to beginning the study.

### 2.3. Hypertrophic Resistance Training Session

The R_T_ sessions comprised six exercises per session targeting major muscle groups of the body (flat barbell press, barbell military press, wide grip lateral pulldown, seated cable row, barbell back squat, and machine leg press). The routines for each session included 3 sets of 10 RM per exercise. Subjects rested 2 min between sets. A standardized warm-up of 15 min was completed at the beginning of each session. Participants were instructed to rest 72 h between training sessions and to refrain from performing any additional resistance-type or high-intensity anaerobic training throughout the duration of the study.

### 2.4. Blood Measurements

After each training session, venous blood samples were taken for determination of hormones (testosterone, growth hormone, and cortisol), cytokines (IL6, IL10, and TNFα) and miR-378. The basal condition was established from a blood analysis collected 2 days prior to the first training session after 48 h of abstention from structured exercise. Blood extractions were performed at the same altitude condition of the corresponding session by specialized staff. Both training centers had available extraction rooms next to the training area.

Immediately following the training session, the antecubital vein of the arm of each participant was canalized via a catheter. The catheter remained permeable by using physiological saline solution. Five milliliters of blood was extracted at minutes 5, 10, 15, and 30 post-exercise. An amount of 2 mL of blood before each extraction was discarded to avoid dilution of the sample. In all cases, blood samples were kept at cold conditions and centrifuged in the following 4 h during 10 min at 3000 rpm. Finally, 500µl aliquots were stored at −70 °C until use.

Hormonal values were assessed at minutes 5, 10, 15, and 30 post-exercise to determine recovery peak values. Analyses were performed in a COBAS C-311 System (Roche, Basel, Switzerland). Cytokine analyses were assessed at minutes 15 and 30 of the recovery by multiple immunoassay kits (Procarta Multiplex immunoassay kit, Invitrogen, Carlsbad, CA, USA), according to the manufacturer’s instructions. Quantitative data were obtained using the Luminex-200 system (Luminex Corporation, Austin, TX, USA), and data analysis was performed on Luminex 100™ IS v2.3 software. Assay sensitivity for each cytokine measured was (pg/mL): IL-10: 0.2466, IL-6: 3.74 and TNFα: 0.835. Finally, cell-free total RNA—primarily miRNA—was obtained at minute 30 of recovery by miRNeasy Serum/Plasma Kit, according to the manufacturer’s instructions (Qiagen, Hilden, Germany). A panel of three invariant miRNAs, two snoRNAs (SNORD95, SNORD96A) and one snRNA (RNU6-2) were used to normalize for variability in sample loading and real-time RT-PCR efficiency [20,21]. Cycling was performed under standardized conditions with 2x QuantiTect^®^ SYBR Green PCR Master Mix on a CFX96 Real-Time PCR Detection System (Biorad, CA, USA). To determine the efficiency of RNA extractions and/or the presence of inhibitors in cDNA synthesis or in PCR, the levels of the Spike-in Control were quantified (C. elegans miR-39 miRNA mimic) and added before RNA extraction by real-time PCR. The fold changes of candidate miRNAs expression were calculated by the equation 2^−ΔΔCt^. All analyses were performed at normoxia using the same equipment.

### 2.5. Statistical Analyses

Data are presented as mean ± standard deviation (SD) or mean standard error (SEM). Normal distributions of the data were confirmed using a Shapiro–Wilk test. Cytokines values were dichotomized to 1 or 0 as detected or non-detected data, respectively, with respect to the minimum detectable threshold established by the analysis kit. Differences between conditions (N vs. H) and time (basal vs. min 15 vs. min 30) were interpreted through the McNemar test. A paired-sample *t*-test was used to assess the session effect (basal vs. maximal value) between exercise conditions (N vs. H) in the peak value of the hormones studied. To quantify the magnitude of the change, standardized differences (i.e., Cohen’s d effect sizes) were also calculated as the mean change divided by the pooled standard deviations in all dependent variables. Threshold classifications were set as follows: >0.2 (small), >0.6 (moderate), >1.2 (large), and >2 (very large).

The relationship between the miRNA response and the higher value of the hormones variables was calculated through a Pearson or Spearman correlation coefficient (r) in both conditions. The corresponding Fisher’s Z-transformed *r* coefficient was used for N and H comparison by calculating the Fisher’s F distribution. Previously, the Spearman’s coefficients (rho) were converted to Pearson’s coefficients (r) when appropriate. For our sample size (n = 13), significance was determined as follows: F > 2.69 [*p* = 0.05] and F > 4.16 [*p* = 0.01]. The inference analysis was determined by the Cohen’s Q effect size calculation (magnitudes were interpreted as follows: <0.1: No effect; 0.1 to 0.3: Small effect; 0.3 to 0.5: Intermediate effect; >0.5: Large effect). A binary logistic regression was carried out to define the relationship between miRNA and cytokines. An indication of the fit of the model was given by Cox-Snell’s R^2^ and an Omnibus test (Chi-Square). All analyses were performed using the software package SPSS (version 26.0, IBM Corp. IBM SPSS Statistics for Windows, Armonk, NY, USA). Effects were considered significant at *p* ≤ 0.05.

## 3. Results

**Figure 2:**
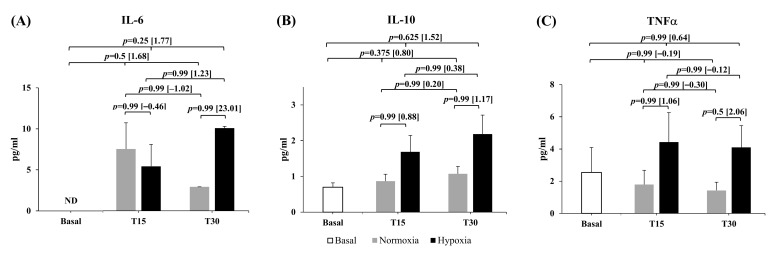
Analysis of the distribution of detected vs. non-detected values of IL-6 (**A**), IL10 (**B**), and TNF-alpha (**C**) in N and H through the 30 min of recovery period. Inference analysis for detected cytokines. Blood samples were taken at rest and 15 min (T15) and 30 min (T30) after exercise in both N and H conditions. Mean and SEM was represented only for detected signal. *p*-value (*p*) and effect size [ES] are represented for each variable. ES was expressed as H-N or post-pre divided by pooled standard deviation; ND: Non-detected.

Cytokine results are displayed in Figure 2. The kit used for these analyses proved not to be sensitive enough to determine interleukin changes in healthy trained athletes (i.e., the threshold for a detected value was too high so that changes that may have occurred went undetected). Thus, we employed a non-parametric dichotomous statistical analysis (McNemar test) on these measures to compare detected vs. non-detected values (i.e., compare the change from 0 to 1 or 1 to 0 from pre- to post-exercise in both environmental conditions). The McNemar test did not show statistical significance at any condition or recovery time in the three cytokines analyzed. However, large increases were observed in the detected IL-6 values after the training session at both environmental conditions (ES = 1.68 and ES = 1.77 for N and H, respectively). The highest detected IL-6 value was reached earlier in N (min 15) compared to the H condition (min 30) (mean ± SEM: min 15 [7.55 ± 3.21 vs. 5.44 ± 2.68 pg/mL]; min 30 [2.93 ± 0.01 vs. 10.10 ± 0.22 pg/mL] for N and H, respectively) (Figure 2A). Moderate to large increases in IL-10 also were observed after R_T_ in N (ES = 0.80, IC [−0.39; 1.98]) and H (ES = 1.52, IC [0.17; 2.88]) (Figure 2B). The magnitude of the change displayed larger peaks values in IL-10 throughout the 30 min of recovery in H (1.02 ± 0.19 vs. 2.31 ± 0.44 pg/mL, for N vs. H, respectively maximum values). The TNFα reached the maximal value in min 15 of the recovery in both conditions although moderate increases favoring H were also depicted (ES = 1.01, IC [−1.18; 3.21] pg/mL) (Figure 2C).

**Figure 3:**
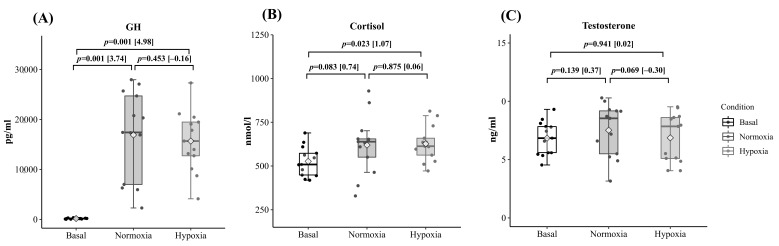
Comparison of the training session effect on maximum post-exercise circulating growth hormone (GH-(**A**)), cortisol (**B**), and testosterone (**C**) in N and H conditions. Data are presented as median, 25th and 75th percentile, and maximum and minimum values. The point clouds are also included. The *p* values are displayed as differences between basal and the corresponding training session. Effect size [ES] is calculated as the mean change (H-N) or (post-pre) divided by the pooled standard deviation.

Figure 3 shows the hormonal results. Cortisol (C) and growth hormone (GH) significantly increased after the training session (Figure 3A,B). Although no statistical differences between environmental conditions were found in the three hormones studied, testosterone displayed a tendency to be reduced post-exercise in H compared to N (7.52 ± 2.25, 6.87 ± 2.09 ng/mL for N and H, respectively; *p* = 0.06) (Figure 3C).

**Figure 4:**
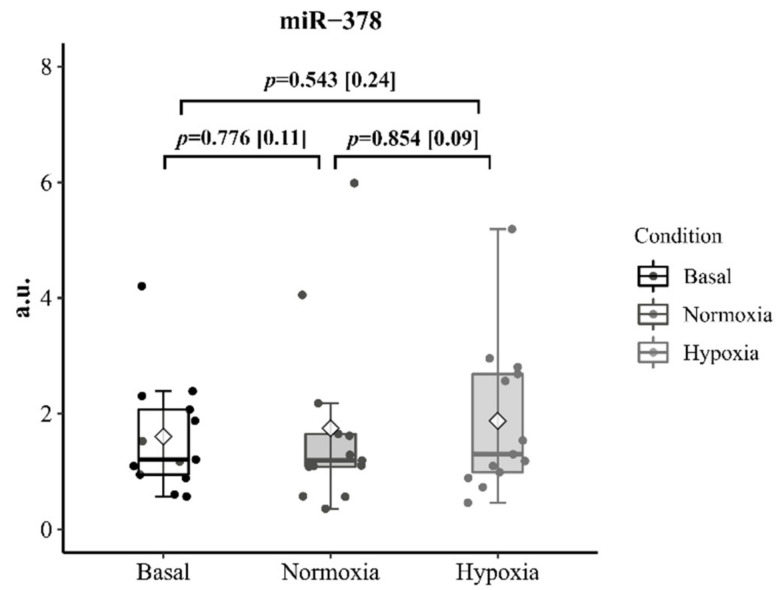
Comparison of the training session effect on maximum post-exercise circulating miR-378 in N and H conditions. Data are presented as median, 25th and 75th percentile, and maximum and minimum values. The point clouds are also included. The *p* values are displayed as differences between basal and the corresponding training session. Effect size [ES] is calculated as the mean change (H-N) or (post-pre) divided by the pooled standard deviation.

Neither environmental condition affected the circulating level of miR-378 after R_T_ (*p* = 0.78, *p* = 0.54 for N and H, respectively). Trivial non-significant increases were achieved after the 30-min recovery period in both conditions (1.75 ± 1.58 vs. 1.88 ± 1.32 arbitrary units in N and H; *p* = 0.854; 95%IC [−1.33; 1.59]; ES = 0.09) (Figure 4).

The correlation analysis displayed only a moderate association between miR-378 with GH and C in N and H (GH: r = 0.654; *p* = 0.03 and r = −0.591; *p* = 0.05; C: r = 0.61; *p* = 0.03 and r = 0.75; *p* = 0.005 respectively) (Table 1). However, a large effect of the environment on the relationship with miR-378 was observed with GH and testosterone. The logistic regression between cytokines and miR-378 did not show a significant relationship in N (IL-6: R^2^ = 0.20; *p* = 0.637; IL-10: R^2^ = 0.40; *p* = 0.116 and TNFα: R^2^ = 0.21; *p* = 0.174) and H (IL-6: R^2^ = 0.11; *p* = 0.279; IL-10: R^2^ = 0.01; *p* = 0.839; and TNFα: R^2^ = 0.19; *p* = 0.241).

## 4. Discussion

The aim of this study was to investigate the influence of the acute ascent to a moderate hypoxic environment on the response induced by a hypertrophy-oriented R_T_ session on inflammatory, hormonal, and miR-378 markers. Exercise increased maximal values in GH and C, while testosterone and miR-378 remained similar to those observed under basal conditions. The observed post-R_T_ response on inflammatory markers suggests an intensification of these cytokines in N and H; however, differences did not rise to statistical significance due to the lack of sensitivity of the analysis kit, limiting our ability to draw a strong conclusion on this outcome. The magnitude of the change in the detected values also seems to indicate a hypoxia-related effect on IL-6, IL-10, and TNFα release into the bloodstream throughout the recovery period. Moreover, IL-6 displayed a larger, albeit delayed, increase in H compared to N. Despite the lack of changes in circulating levels of miR-378, the moderate association with GH and C agrees with the research hypothesis and may indirectly indicate a relationship with the expression of other target genes involved in muscle hypertrophy [4].

The results displayed a rapid R_T_-induced inflammatory response, which has been attributed to an acute potentiation of myogenesis through the circulating TNFα and IL-6 [14]. The magnitude inference analysis of the results showed a delay in the increase of IL-6 in H compared to N after exercise. IL-6 is both a pro- and anti-inflammatory cytokine [22], released in an exponential fashion into the bloodstream in response to damaging exercise and depletion of muscle glycogen stores [23]. It can be hypothesized that the greater stabilization in pH observed in N in several studies [6,19] allows greater glucose resynthesis [24], which may explain the early increase in IL-6 in this condition and the limited TNFα production, highly dependent on O_2_ availability. Conversely, it is known that during exercise in H, an increase in reactive oxygen species (ROS) associated with the rise in the expression of hypoxia-inducible factor-1 (HIF-1) [25], in conjunction with the high buffering activity that limits the ability to resynthesize glucose [19], could help to explain the progressive and delayed increase in IL-6 through TNFα overexpression [26,27]. An increase in circulating IL-6 potentially enhances anabolic signaling via STAT3, which is required for the increase in the myogenic regulatory factors (such as MyoD), as well as by inhibiting the apoptotic pathways of TNFα through SOCS1 expression (Figure 1) [15,22,28]. As has been observed in other studies [14], the net IL-6 production is intensified in H. The progressively larger increases in IL-10 release throughout the recovery period with respect to the N values achieved could also emphasize this response [10,29]. Moreover, although a positive association between IL-10 and C levels was expected, it was not observed, probably due to the aforementioned limitation in the IL-analysis procedure. The present results failed to clearly show that the same R_T_ session performed under H conditions enhances the hormonal, inflammatory and circulating miR-378 responses compared to normoxia. However, it is possible that hypoxia may prolong the duration of time that various cytokines circulate post-exercise compared to N, which potentially could enhance the hypertrophic response.

R_T_ produced large post-exercise increases in circulating GH and C, although altitude did not statistically affect the magnitude of their peak values. This may be explained by the similar metabolic stress response (i.e., maximal blood lactate and serum Ca^2+^) observed for both environmental conditions [19]. Consistent with this hypothesis, C dynamics reflect the global stress and metabolic requirements [30]. GH release also seems to be mediated by blood lactate and H^+^ levels [1,31]. However, despite the general increase in lactate production during hypoxic R_T_ described in the literature [1,7,8], some studies have failed to observe statistical differences in peak lactate [19] and GH levels after R_T_ under hypoxic conditions compared to N [32]. The type of hypoxia alters the physiological response of systems, such as the ventilatory system, affecting the behavior of variables sensitive to changes in the ventilatory pattern [33]. Therefore, the hypocapnic response throughout the initial hours of exposure to terrestrial hypoxia found in other studies [19,34] seems to increase the pre-exercise pH and potentially helps to explain the physiological reduction of GH release during acute exercise at terrestrial H vs. at normobaric hypoxia [35].

The results do not reveal a significant change in circulating testosterone after the acute hypertrophic strength session and hypoxia does not seem to affect it [19,36]. Although H revealed a small decrement in the peak circulating post-exercise testosterone response compared to N, the absence of differences in this hormone with respect to basal conditions when contrasted with the changes in GH and C blood values, highlights the complexity of the acute hormonal response to strength exercise.

In addition to the inflammatory and hormonal response, the effects of hypoxia on miR-378, which is linked to protein synthesis regulation, potentially could elucidate the miRNA relationship with other muscle building agents in trained individuals [16]. The overexpression of miR-378 has an inhibitory effect on its target gene expression that, in some way, regulates the processes that lead to muscle hypertrophy by allowing the MyoD factor to be expressed, as also IL-6 does [14]. Contrary to our expectations, circulating miR-378 levels showed similar values between basal conditions and after acute R_T_ in N (*p* = 0.776; 95% IC [−1.236¸0.945]) and H (*p* = 0.543; IC95% [−1.214; 0.067]) and did not display any relationship with IL-6. Under N conditions, increases in circulating miR-378, miR-21, and miR-940 are described immediately after acute aerobic exercise in chronic heart failure patients [37]. To our knowledge, only one study [16] has investigated this outcome in healthy individuals during R_T_ although, unlike in our study, the researchers measured the expression of miR-378 and other miRNAs from muscle biopsies rather than serum samples. Davidsen et al. [16] observed elevations in miR-378 expression and reported a positive relationship between the abundance of this miRNA and muscle mass gains after a 12-week R_T_ program. miR-378 displayed a downregulation in those classified as low responders for lean mass gains (linked to a reduced miRNA profile for IGF-1), whereas high responders exhibited no changes from baseline to post-exercise. The fact that miR-378 is not expressed specifically in muscle tissue could explain its relevance as a biomarker of exercise adaptations in clinical populations [37] and with sedentary individuals [16], while limiting its applicability to trained individuals. Thus, the variability between subjects could corroborate the presence of responding and non-responding participants. This hypothesis warrants further investigation.

Hypoxia seems to produce transient increases in IGF-1 mRNA after a R_T_ [14]_._ Currently, 3 different IGF1 isoforms are described, all of them expressed in muscle tissue. In particular, the IGF1-Ec isoform, also known as mechano growth factor (MGF), is expressed immediately post-exercise to facilitate the repair of local muscle damage through a signaling pathway that includes mTOR (PI3K-Akt-mTOR) and other calcium-dependent pathways. The link between GH and IGF1 is also documented in the literature [38]. Our study did not measure IGF1, but the relationship between GH and miR-378 observed in N could indirectly indicate the activation of the protein synthesis pathway linked to the miR-378 response. The association observed between GH and miR-378 is consistent with our research hypothesis, although the weakness of the association, as well as the absence of a relationship with other markers related to muscle growth (such as testosterone or IL-6) raises skepticism as to potential conclusions. The interaction displayed between H and GH [35], and also indirectly with testosterone (Table 1), could conceivably help to explain the H effect on the association between miR-378 and these hormones. However, the absence of activation of the mTOR pathway after an isolated R_T_ session [39] at acute hypoxia may question the influence of the IGF1, GH, and mR-378 activation pathway in the early adaptations of skeletal muscle to resistance exercise. Longer duration studies are required to clarify the role of these mediators in muscle hypertrophy in response to R_T_ in hypoxia.

Several limitations of this study should be noted: (1) The duration of the post-exercise recovery period lasted only 30 min, thus limiting the ability to draw conclusions beyond this acute time frame. (2) A double-blind design could not be employed due to the use of a terrestrial altitude; to reduce the potential for confounding effects, participants were not informed about the expected altitude effect. (3) Although the McNemar test showed no differences in the three cytokines studied in either the exercise or environmental conditions, limitations in the sensitivity of the test employed potentially could mask the ability to detect positive results. Magnitude-based inferences analysis were used to provide practical insights into interleukins results. Further studies should be performed using commercially available kits with higher sensitivity. (4) Our sample size was relatively low, which may have compromised the ability to detect statistical significance in some outcomes.

## 5. Conclusions

In conclusion, a traditional hypertrophic R_T_ program performed at acute moderate hypoxia does not seem to provide a more potent hormonal or inflammatory response that could enhance the muscle growth stimulus to a greater extent than equivalent training in normoxia. However, the standardized mean difference values in the myokines studied point to an intensification of the inflammatory response in H that should be further investigated in relation to the assessment of athletes’ health and the role they have on activating muscle adaptations to strength training. Although the acute muscle adaptative response to this type of R_T_ seems not to be mediated by changes in circulating levels of miR-378, the link observed with the GH and C could indirectly indicate the miR-378 relationship with the expression of other target genes involved in R_T_-induced muscle hypertrophy. The response observed cannot necessarily be extrapolated to longer time periods and types of hypoxia (e.g., terrestrial vs. simulated).

## Figures and Tables

**Table 1:** Relationship between the miRNA-378 response and the peak value of the hormone’s serum concentrations in both altitude condition.

	miR-378				N vs. H		
	N		H				
	*r*	*p*	*r*	*p*	Z	*p* [F > 2.69] = 0.05 *p* [F > 4.16] = 0.01 [%95 IC]	Q
GH (pg·mL^−1^)	**0.654**	**0.026**	−0.591	0.051	−4.621	***p*= 0.01** [−2.081; −0.841]	1.461 Large
Testosterone (ng·mL^−1^)	0.462	0.128	−0.456	0.133	−3.139	***p*= 0.05** [−1.613; −0.373]	0.993 Large
Cortisol (nmol·l^−1^)	**0.606**	**0.035**	**0.747**	**0.005**	0.833	- [−0.356; 0.883]	0.263 Small

*r*: Pearson correlation coefficient; *p*: *p*-value; Z: value of the statistic in the Fisher’s Z-transformed r coefficient comparison; F: value of Fisher’s F distribution only for *p* = 0.05 or *p* = 0.01. Non-significant values are not expressed; Q: Cohen´s q effect size; N: normoxia; H: hypoxia; miR: microRNA.

## Data Availability

All available data are included in the manuscript. It is not necessary to refer to any additional information.
